# Genetic and Molecular Epidemiological Characterization of a Novel Adenovirus in Antarctic Penguins Collected between 2008 and 2013

**DOI:** 10.1371/journal.pone.0157032

**Published:** 2016-06-16

**Authors:** Sook-Young Lee, Jeong-Hoon Kim, Tae-Kun Seo, Jin Sun No, Hankyeom Kim, Won-keun Kim, Han-Gu Choi, Sung-Ho Kang, Jin-Won Song

**Affiliations:** 1 Department of Microbiology, College of Medicine, Korea University, Seoul, Republic of Korea; 2 Division of Life Sciences, Korea Polar Research Institute, Incheon, Korea; 3 Department of Pathology, College of Medicine, Korea University, Guro Hospital, Seoul, Korea; 4 Division of Polar Ocean Environment, Korea Polar Research Institute, Incheon, Korea; French National Centre for Scientific Research, FRANCE

## Abstract

Antarctica is considered a relatively uncontaminated region with regard to the infectious diseases because of its extreme environment, and isolated geography. For the genetic characterization and molecular epidemiology of the newly found penguin adenovirus in Antarctica, entire genome sequencing and annual survey of penguin adenovirus were conducted. The entire genome sequences of penguin adenoviruses were completed for two Chinstrap penguins (*Pygoscelis antarctica*) and two Gentoo penguins (*Pygoscelis papua*). The whole genome lengths and G+C content of penguin adenoviruses were found to be 24,630–24,662 bp and 35.5–35.6%, respectively. Notably, the presence of putative sialidase gene was not identified in penguin adenoviruses by Rapid Amplification of cDNA Ends (RACE-PCR) as well as consensus specific PCR. The penguin adenoviruses were demonstrated to be a new species within the genus *Siadenovirus*, with a distance of 29.9–39.3% (amino acid, 32.1–47.9%) in DNA polymerase gene, and showed the closest relationship with turkey adenovirus 3 (TAdV-3) in phylogenetic analysis. During the 2008–2013 study period, the penguin adenoviruses were annually detected in 22 of 78 penguins (28.2%), and the molecular epidemiological study of the penguin adenovirus indicates a predominant infection in Chinstrap penguin population (12/30, 40%). Interestingly, the genome of penguin adenovirus could be detected in several internal samples, except the lymph node and brain. In conclusion, an analysis of the entire adenoviral genomes from Antarctic penguins was conducted, and the penguin adenoviruses, containing unique genetic character, were identified as a new species within the genus *Siadenovirus*. Moreover, it was annually detected in Antarctic penguins, suggesting its circulation within the penguin population.

## Introduction

Adenoviruses (family *Adenoviridae*) are non-enveloped, double-stranded DNA viruses with genomes ranging in size from 26 to 45 kbp. Adenoviruses infect the respiratory tract, eyes, gastrointestinal tract, and several other organs, causing gastroenteritis and respiratory disease in many species [1]. The *Adenoviridae* family comprises of five genera: *Mastadenovirus*, *Aviadenovirus*, *Atadenovirus*, *Siadenovirus*, and *Ichtadenovirus* [2]. *Mastadenovirus* has been identified in mammalian species such as human, monkey, dog, cattle, swine, mouse, and bat [3–9]. *Atadenovirus* has been isolated from wide range of hosts, including reptiles, birds, and mammals [10–12]. *Aviadenovirus* and *Ichtadenovirus* have been detected in bird species and fish, respectively [13, 14]. Viruses of the genus *Siadenovirus* have been found in amphibian, bird, and reptile hosts [15–23]. Recently, the Chinstrap penguin adenovirus 1 (CSPAdV-1), which belongs to the genus *Siadenovirus*, was discovered in dead Chinstrap penguins (*Pygoscelis antarctica*) collected from Antarctica [24].

Antarctica has been isolated for long periods because of its geographical and climatic conditions. However, global warming, animal behavior, and human activities in Antarctica implied the potential possibilities of introduction and spread of infectious disease [25–27], and the circumstantial evidences of several viral infections in Antarctic avifauna were reported, such as, adenoviruses in South Polar skuas (*Catharacta maccormicki*) and Chinstrap penguins and papillomavirus, influenza A virus, and polymavirus in Adelie penguins (*Pygoscelis adeliae*) [21, 24, 28–30].

Here, we characterized the whole genome of the novel penguin adenoviruses as a further study of partial CSPAdV-1 [24] and examined the molecular epidemiology of these viruses in Antarctic penguins, during 2008–2013.

## Materials and Methods

### Samples

Seventy-eight carcasses of penguin were collected from the vicinity of the King Sejong station, Narębski Point, and Ardley Island, located on the King George Island, Antarctica, during 2008–2013, by permission in Ministry Foreign of Affairs of Republic of Korea. The penguins were composed of 30 Chinstrap penguins (CSP), 46 Gentoo penguins (*Pygoscelis papua*, GP), and 2 Adelie penguins (AP). No pathognomonic signs were observed in necropsy finding. Internal samples (from the lung, liver, kidney, heart, intestine, trachea, spleen, brain, lymph node, wounded-bill, and feces) of the all penguin were collected after dissection, and stored at -70°C until used.

### PCR and DNA Sequencing

Total DNA was extracted from pooled internal samples using High Pure PCR Template preparation kit (Roche, Indianapolis, IN, USA) according to the manufacturer’s instructions. The primer pairs specific for penguin adenovirus were used for entire genomic sequencing (Table 1), and primers Ad_hex1514F (5’-ACATTCAGGTTCCTCAGA-3’), Ad_hex2963R (5’-TTAT(A/G)C(C/T)GAAGCAGTTCCA-3’), Ad_hex2140F (AGTCAGTCTAATATGAC-3’), and Ad_hex2753R (5’-GAAGAGTTCCAGTAGC-3’) were used for molecular epidemiological survey of adenoviral infection in penguin population. The presence of sialidase gene was tried to be verified by PCR using the primers Ad_ITR (CAATCAAAATTGATACCGCATGT), Ad_hyd112R (TCAGCAACAGCTCTGGCA), and Ad_hyd134R (AGCCATAGTACGCTTAGCA). The final PCR volume of 50 μl was composed of 10 mM dNTP, 10 pmol/ml of forward and reverse primer, 0.25 unit of TaKaRa Ex Taq (TAKARA BIO INC. Shiga, Japan), and 50 ng of template DNA. PCR was performed under the following conditions: 1 cycle of 95°C for 5 min, followed by 14 one degree step-down cycles, each consisting of denaturation at 95°C for 40 s, with annealing from 50–37°C for 40 s, and extension at 72°C for 1–2 min. This was followed by 25 cycles consisting of denaturation at 95°C for 40 s, annealing at 42°C for 40 s, and extension at 72°C for 1–2 min, and finally, at 72°C for 5 min in a Mastercycler (Eppendorf, Germany). Extension time was altered according to the expected product size. The amplified product was purified by PCR Purification Kit (QIAGEN, Chatsworth, CA) and sequenced by Big Dye 3.1 terminator cycle sequencing reagents on ABI 3730 Automated DNA sequencer (Applied Biosystems, Forest City, CA). The inverted terminal repeat (ITR) sequences of adenovirus were confirmed by modified-RACE (Rapid Amplification of cDNA Ends, TAKARA BIO INC. Shiga, Japan).

**Table 1:** The list of primers used for whole genome sequencing of penguin adenoviruses.

Gene	Primer	Sequence (5’-3’)	Polarity
	ITR_EcoR	GGA TCC CAA TCA AAA TTG ATA CCG	+/-
IVa	IVa518F	ATT CAT CAA GTA CAA CAC A	+
	IVa626R	AAT GTG CCT TTG ATG ATG T	-
Polymerase	pol560F	AAT AAG CTG TCT GTA TCT CC	+
	pol658R	TAT GAG TGA TTG GACAGA	-
	pol1439F	TGA TAG CTT GCT TGT TCT CTG	+
	pol2132R	CAA ATH ACA CAC ACA TCT CT	-
	pol2358F	TGT GTG GCT AAT AGA ATC TCA	+
	pol2973R	ATG GAT CAA ACA ACA AAA ATG	-
pTP	pTP180F	TTG GAT CTC CAG ATT CTG	+
	pTP321R	ATC TCC ATT CCT CAG A	-
	pTP1474F	ART TTC THG GTC TTC T	+
52K	52K189R	AGA TTA TTT TGA GGC A	-
	52K798F	TGT GTC ARA TWG GAG T	+
pIIIa	pIIIa42R	AGA CAA AAT TGG AGC A	-
Penton	pen_EcoR	GAA TTC ATG CAG TCT TCA ACT CCA	+
	pen250R	CCC AYC TWG ACC TAT CAT CT	-
	pen1233F	AGG AGT TCA AAG AGT GGT	+
pVII	pVII126R	CGA ACC CTA ACA GTA TA	-
Hexon	hex122F	TAA TAA GAA GTT TAG AGA	+
	hex365R	TTG TAT GCT GTC CCG CCA T	-
	Hex1514F	ACA TTC AGG TTC CTC AGA	+
	hex1693R	TGC TTG CTC CAT CCTTCC TA	-
	hex2583F	CAG AGT TTG CTG TA	+
	hex2753R	GAA GCA GTT CCA GTA GC	-
DBP	DBP162F	TTT TCA TTG CCT GCCTCA GA	+
	DBP448R	AAA GAT GAT AAT CGT GAC CA	-
100K	100K290F	CAC TTA AGG AGA CAA GCA	+
	100K620R	ACT ATG ACA AGT CTG CT	-
	100K1615F	CGG AGA TTA TCA GCA GA	+
	100K1871R	TCC AAA TAG ATA CCA GAA CCT	-
pVII	pVII510F	TGG CAA TGA GT CTC CCA	+
	pVII619R	AAT ACC GGA TTC TGT CCT	-
Fiber	Fiber_EcoR	GAA TTC ATG GAA TCA CCA AGA AAG	+
	Fiber423R	TCC TGC AGA ATC AGT TGT	-
	FO64F	AAA AAC ATC AGG TGC GTG T	-
	end23486F	TGC CTA CAT ATA CAT TCA GCA	+
	end24279R	TGG TTT ATG TAC CCT TGA CT	-

### Phylogenetic analysis

The phylogenetic analysis was carried out based on the DNA polymerase and hexon sequence of penguin adenoviruses. Sequences of adenoviruses were retrieved from the GenBank. Multiple alignments of adenoviral sequences were generated by Clustal W method in MegAlign of DNAstar (Lasergene program version 5, DNASTAR Inc. Madison, WI). Phylogenetic trees were generated by a Bayesian inference of phylogeny throughout the MrBayes V3.1.2 software [31, 32] and Maximum likelihood (ML) in methods of MEGA6.0 (Molecular Evolutionary Genetics Analysis 6.0) software [33]. WAG and GTR model contributed to approximate the posterior probabilities (pp) of trees inferred from amino acid and nucleotide alignments, respectively. The topologies of ML trees were evaluated by a bootstrap analysis of 1,000 iterations by using MEGA 6.0.

## Results

### Genetic character and genome organization

The whole genomes of 2 Chinstrap penguins (CSPAdVno3 and CSPAdVno4, GenBank accession no.KP144329 and KP144330) and 2 Gentoo penguins (GPAdVno4 and GPAdVno5, KP279746 and KP279747) collected in 2010 were sequenced. The genome lengths were 24,662 bp (CSPAdVno3), 24,659 bp (CSPAdVno4), 24,630 bp (GPAdVno4), and 24,633 bp (GPAdVno5). The G+C contents of the complete genomes were 35.5% in CSPAdV, and 35.6% in GPAdV. The G+C content of each gene ranged from 30.6–47.1%; the gene of the histone-like core protein precursor pVII was found to have the highest G+C content.

**Fig 1:**
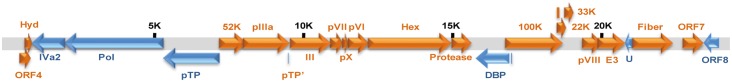
Putative genetic content of penguin adenovirus. The range of entire genome lengths of penguin adenoviruses were 24,630–24,662 bp, and the putative sialidase gene was not detected in the penguin adenovirus genomes. The genomic lengths are indicated at5,000 bp intervals.

The genetic content and structure of penguin adenovirus are presented in the schematic genome map in Fig 1, and contains 23 ORFs. The ORF4 reported in TAdV-3 and raptor adenovirus 1 (RAdV-1) was also discovered in penguin adenovirus, but the sialidase gene existing between the inverted repeat (ITR) region of the 5’ end and ORF4 was not detected in the penguin adenovirus genomes by modified RACE-PCR. The bi-directional analysis of ORF in the left-hand end of the genome between ITR of the 5’ end and initial hydrophobic protein (hyd) showed only putative ORF4 (360 bp), as ORF is longer than 200 bp.

The lengths of most of the genes were identical among the various penguin adenoviruses, but a few genes, such as hexon and E3 gene, showed different lengths between CSPAdV and GPAdV (Table 2). A lack of 3 nucleotides (amino acid residue G; CAG, at nucleotide positions 722–724) in the hexon gene of CSPAdVno4 and GPAdVno4 [24], and an absence of 21 nucleotides (amino acid residues DGTYPFS: GATGGAACTTACCCCTTTTCT, nucleotide positions 445–465 in CSPAdV) in the E3 gene of GPAdV were identified. Moreover, there was a lack of several nucleotides in the noncoding region of CSPAdV, specifically at position 22,750–22,760 (AAATTATAGAC; on the basis of the GPAdV sequence) located between fiber and ORF7. The sequence of GPAdV was also shown to be shorter by 11nt (GCTAGTATAAA, nucleotide position 342–356 in CSPAdV) in noncoding region, downstream of ORF4 and 13 nt (CTGTTTGGTACAA, nucleotide position 22,847–22,859 in CSPAdV) in noncoding region, immediately before ORF7 (Table 2).

**Table 2:** Summary of deletions of nucleotides and their position in the entire sequence of Chinstrap penguin adenovirus (CSPAdV) and Gentoo penguin adenovirus (GPAdV).

Viruses	Region (position)	Deficient nucleotide sequences
GPAdV	Noncoding region in downstream of ORF4 (346-56nt)	GCTAGTATAAA
CSPAdVno4 and GPAdVno4	Hexon (724-726nt)	CAG
GPAdV	E3 (455-465nt)	GATGGAACTTACCCCTTTTCT
CSPAdV	Noncoding region between fiber and ORF7 (22,750–22,760nt)	CTGTTTGGTACAA
GPAdV	Noncoding region, immediately before ORF7 (22,847–22,859nt)	AAATTATAGAC

The lengths of the ITR sequences of CSPAdV and GPAdV were identical (i.e., 30 bp), and a single nucleotide difference at position 24 (C/T) was detected between CSPAdV and GPAdV.

### Identification of existence of sialidase gene

The absence of sialidase gene in penguin adenovirus was verified from the 4 completely sequenced penguin adenoviruses, CSPAdVno3, CSPAdVno4, GPAdVno4, and GPAdVno5, by modified RACE-PCR. The 22 penguins that were detected with adenoviral genome were further tested for the presence of sialidase gene by PCR using the specific primer set, the primers from hyd gene and ITR region of 5’ end (ITR/hyd). The size of PCR products by ITR/hyd was identified to be approximately 850 bp in the 19 penguins including the 4 penguin adenoviruses that were completely sequenced (data not shown), and the PCR results in the 3 penguins were negative.

### Phylogenetic analysis

**Fig 2:**
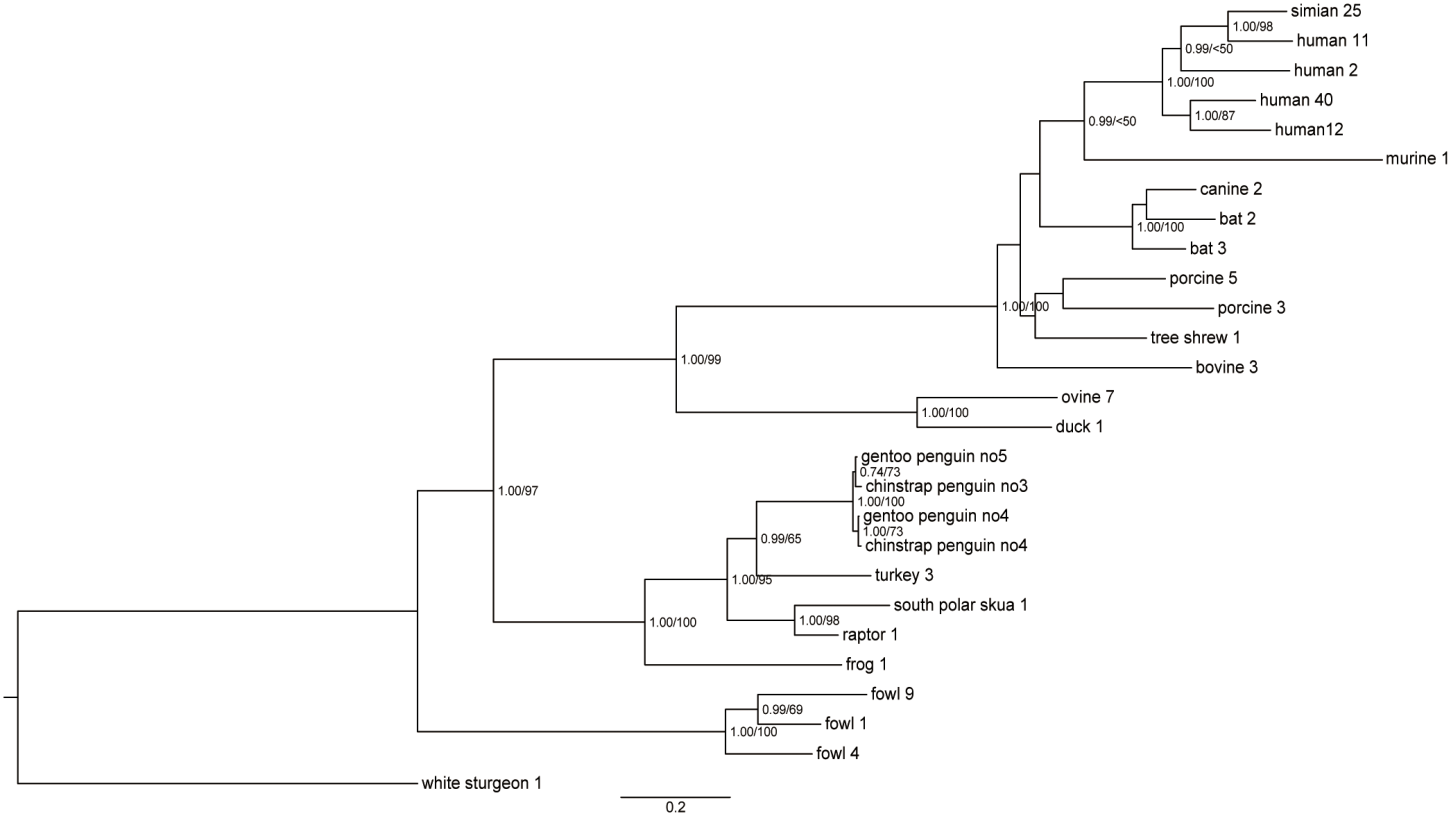
Phylogenetic analysis of amino acid sequences of penguin adenoviral hexon. The phylogenetic tree, based on entire hexon genome sequences of penguin adenoviruses, was generated using the Bayesian and maximum likelihood (ML) method. The first number indicates the Bayesian posterior probability, and the second number indicates the ML bootstrap value as a percentage. Scale bars indicate the number of nucleotide substitutions per site.

**Fig 3:**
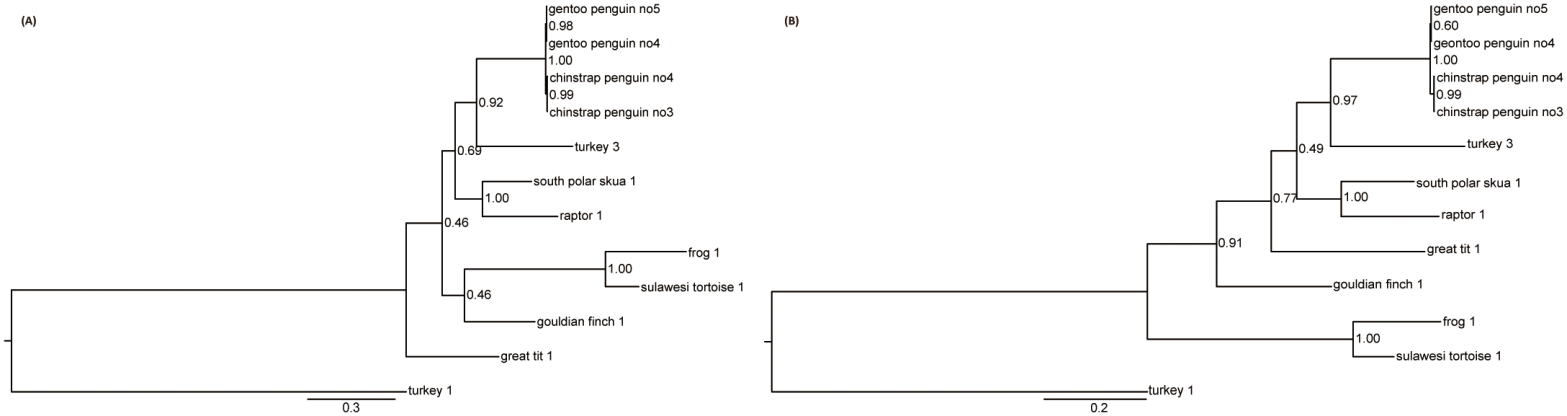
Phylogenetic relationships within the genus *Siadenovirus* based on partial DNA polymerase sequences (a) inferred from nucleotide alignment and (b) from amino acid alignment. The phylogenetic tree was obtained with a Bayesian inference of phylogeny by the MrBayes v3.1.2 software.

Phylogenetic analyses of entire hexon using the Bayesian and ML methods indicated that penguin adenoviruses clustered significantly with *Siadenovirus* sp., as supported by the high posterior probabilities and bootstrap values of 100%. The analysis of the amino acid sequence of entire hexon showed the closest relationship and sharing of ancestor with TAdV-3,with high posterior probabilities (pp value 0.99) (Fig 2). The sister clades within the penguin adenoviruses were constructed by clustering of CSPAdVno3 and GPAdVno5, and CSPAdVno4 and GPAdVno4, respectively. Support for the clade of penguin adenoviral hexon was stronger in the phylogeny by Bayesian than by ML method. Also the phylogenetic analysis of partial DNA polymerase of 274 nt (91 aa) showed that penguin adenoviruses were clustered with the TAdV-3 (> 0.92) (Fig 3). The nucleotide alignment of partial DNA polymerase showed the clustering of Gouldian finch AdV with the Sulawesi tortoise and frog AdV (FrAdV-1) with low pp value (<50) and the first divergent of great tit AdV (Fig 3A), while the calculation based on the amino acid alignment showed the grouping of bird-related siadenoviruses on the same branch (Fig 3B).

The entire hexon sequence of penguin adenoviruses shared 76.1–76.2% (77.2–77.4%, amino acid), 73.9–74.4% (73.4–73.5%), 73.3–73.5% (74.1–76.5%), and 68.5–68.8% (67.5–68.6%) identity with the genomes of TAdV-3, South Polar skua adenovirus 1 (SPSAdV-1), raptor adenovirus 1 (RAdV-1), and frog adenovirus 1 (FrAdV-1), respectively. In addition, the entire DNA polymerase of penguin adenoviruses showed identity of 60.7–70.1% (52.1–67.9%, amino acid) with other siadenoviruses. The hexon and DNA polymerase sequences of penguin adenovirus showed identity of 99.2–100% (99.1–99.9%) and 98.6–99.1% (97.9–99.5%), respectively.

### Molecular epidemiology

To investigate adenoviral infections in the penguin population from 2008 to 2013, we examined 552 internal samples from 78 penguin carcasses by amplifying of a part of the hexon gene. The adenoviral genome was detected in 22 penguins (28.2%, 22/78), including 12 Chinstrap penguins (40%, 12/30), 9 Gentoo penguins (19.6%, 9/46), and an Adélie penguin (50%, 1/2). PCR positivity rate for the adenoviral genome was highest for the Chinstrap penguin population. The adenovirus detection rate was highest in 2008 (100%, 2/2), followed by 2010 (60%, 9/15) (Table 3). Interestingly, of the penguin adenovirus genome detected from various sample types, the PCR-positivity rate was highest in the kidney (63.6%, 14/22), followed by lung samples at 36.4% (8/22), and greater than approximately 11% in the liver, heart, intestine, trachea, spleen, and fecal samples. However, the adenovirus genome was not identified in the lymph node or brain samples (Table 4). The detection rate of the penguin adenovirus genome with respect to geographic location was 20/72 (27.8%) at Narębski Point, 1/1 (100%) near the King Sejong station, and 1/5 (20%) at Ardley Island.

**Table 3:** PCR positivity rate for penguin adenovirus in Chinstrap penguins (CSP), Gentoo penguins (GP), and Adelie penguins (GP), Antarctica, 2008–2013.

Year	PCR-positive/total (%)			Total (%)
	CSP	GP	AP	
2008	-	2/2 (100)	-	2/2 (100)
2009	1/2 (50)	0/1 (0)	-	1/3 (33.3)
2010	5/8 (62.5)	4/7 (57.1)	-	9/15 (60)
2011	-	2/7 (28.6)	1/1 (100)	3/8 (37.5)
2013	6/20 (30)	1/29 (3.4)	0/1 (0)	7/50 (14)
Total (%)	12/30 (40)	9/46 (19.6)	1/2 (50)	22/78 (28.2)

**Table 4:** Detection of the adenoviral genome in tissue and fecal samples collected from penguins infected with adenovirus by PCR.

Sample	PCR-positive samples/total samples from PCR-positive penguins (%) *			Total (%)
	CSP	GP	AP	
Lung	6/12 (50)	2/9 (22.2)	0/1 (0)	8/22 (36.4)
Liver	4/12(33.3)	0/9 (0)	0/1 (0)	4/22 (18.2)
Kidney	6/12 (50)	8/9(88.9)	0/1 (0)	14/22 (63.6)
Heart	3/11 (27.3)	0/8(0)	1/1 (100)	4/20 (20)
Intestine	4/11 (36.4)	1/7 (14.3)	0/1 (0)	5/19 (26.3)
Trachea	4/11(36.4)	0/7 (0)	0/1 (0)	4/19 (21.1)
Spleen	1/7 (14.3)	0/1 (0)	0/1 (0)	1/9 (11.1)
Brain	0/7 (0)	0/3 (0)	0/1 (0)	0/11 (0)
Lymph node	0/1 (0)	-	-	0/1 (0)
Feces	0/6 (0)	2/3 (66.7)	0/1 (0)	2/10 (20)

*All types of tissue and fecal samples were not collected from every penguin. PCR was performed only for the collected samples. Accordingly, the total number of each sample collected from each penguin differs.

## Discussion

### Genetic features and phylogeny of penguin adenovirus

Our previous study suggested that based on the partial hexon gene sequence, CSPAdV merits the establishment as new species in the genus *Siadenovirus* [24]. In this study, the entire genome sequence and structure of GPAdV and CSPAdV were determined. The complete genomes of penguin adenoviruses (24,630–24,662 bp) were substantially shorter than those of other siadenoviruses, including SPSAdV-1 (26,340 bp), RAdV-1 (26,284 bp), TAdV-3 (26,263 bp), and FrAdV-1 (26,163 bp). The G+C content of the penguin adenoviruses (35.5–35.6%) also complied with that of four other siadenoviruses genomes (TAdV-3: 34.9%, SPSAdV-1:34.2%, RAdV-1:38.5%, FrAdV: 37.9%). The low G+C content is a character conserved across all *Siadenovirus* species, and is related with host jumping in adenoviruses [20, 22]. Hence, the diverse host range of siadenoviruses can be attributed to their host switching.

Based on the phylogenetic trees of entire hexon as well as partial DNA polymerase, penguin adenoviruses were included within the genus *Siadenovirus*. In the family *Adenoviridae*, a novel adenovirus species is usually defined as one detected in a new host species and having more than a 15% phylogenetic distance in DNA polymerase protein compared with previously characterized adenovirus species [34, 35]. The DNA polymerase gene showed the differences of29.9–39.3% (32.1–47.9%, amino acid) with *Siadenovirus* species. Furthermore, the penguin adenoviruses discovered from new host species have not been previously reported. Based on these criteria, we concluded that penguin adenoviruses were novel adenovirus in the genus *Siadenovirus*. The close relationship of penguin adenovirus and TAdV-3 was strongly supported by a high pp value (> 0.92) in the phylogenetic analysis of entire hexon gene and partial DNA polymerase. The phylogeny of entire hexon of penguin adenovirus showed the clustering of CSPAdVno4 and GPAdVno4 because of the deletion of an amino acid in hexon gene.

The genetic structure of the novel penguin adenovirus showed the absence of putative sialidase gene. The lengths from 5’ end to ORF4 of penguin adenoviruses are significantly shorter (758–769 bp) than that of other siadenoviruses (2,028–2,142 bp). Moreover, except the ORF4, any other ORF longer than 200 bp, between 5’end and ORF4 was not detected. The sialidase gene, named so due to its similarity to bacterial sialidase gene, is known as a putative gene that is specific to the genus *Siadenovirus*. Although the function of sialidase is still unknown, it may be related to entry in host cell by binding sialic acid residues [20]. The genetic structure of the novel penguin adenovirus showed the absence of putative sialidase gene. Nonetheless, their genetic characters, short genome length, low G+C contents, and phylogeny, indicated that penguin adenovirus belongs to the genus *Siadenovirus*. However, this genomic organization difference, the absence of putative sialidase gene can be seen as a further species demarcation criterion. Therefore, additional studies on the function of sialidase and the presence of sialidase gene in *Siadenovirus* sp. are necessary, since complete sequences are only available for 5species: FrAdV-1, TAdV-3, RAdV-1, SPSAdV-1, penguin AdV 1 (abbreviated as PeAdV-1).

The genetic mutations between virulent and avirulent strain of THEV (Turkey hemorrhagic enteritis virus) has been compared [36] and those in the fibre were studied recently at the 3D level [37], while further missense mutations on sialidase and E3 gene were found only in the virulent strains [36]. Among penguin adenoviruses, the variations of sequences, including the lack of 7 amino acids in E3 gene, were verified, but the biological character affected by the genetic variations could not identified because of failure of the isolation of penguin adenovirus [24]. Further study on the isolation of penguin adenovirus will be required to reveal the biological character.

### Molecular epidemiology and infection of penguin adenovirus

The molecular epidemiological study of penguin adenovirus from 2008–2013 indicated that the infection predominantly affects the Chinstrap penguin population, and the annual detection of penguin adenoviruses suggests their prevalence and circulation in Antarctic penguin populations. However, significant divergence among the different penguin adenovirus sequences from different geographic regions was not detected.

The novel viruses in the genus *Siadenovirus*, Sulawesi tortoise adenovirus 1 and Gouldian finch adenovirus, cause severe systemic infections in most of the organs [22, 23]. In the internal organs of penguins, the adenovirus was detected at a high rate in the kidney in addition to the lung, liver, heart, intestine, trachea, spleen, and feces. These results suggest that the penguin adenovirus causes systemic infections in penguins.

In conclusion, four penguin adenoviruses were identified from two dead Chinstrap penguins and two Gentoo penguins, the endemic species in Antarctica [38, 39]. The penguin adenoviruses were identified as members of a new candidate species, containing unique genetic character, in the genus *Siadenovirus*. In addition, our molecular epidemiological data indicated that the penguin adenovirus is prevalent and circulating in Antarctic penguin populations.
